# Supplementary material for: Phosphomimetic S207D Lysyl–tRNA Synthetase Binds HIV-1 5′UTR in an Open Conformation and Increases RNA Dynamics
Source: Viruses. 2022 Jul 16;14(7):1556. doi: 10.3390/v14071556 (PMC9315659; doi:10.3390/v14071556)
Supplement: Supplementary file 1 [file viruses-14-01556-s001.zip › viruses-1775293-supplementary.pdf]

SUPPLEMENTARY TABLES AND FIGURES

for

Phosphomimetic S207D lysyl-tRNA synthetase binds HIV-1 5′UTR in an open conformation and increases RNA dynamics

William A. Cantara[1,2,3,†,^,*], Chathuri Pathirage[1,2,3,4,†], Joshua Hatterschide[1,2,3,^], Erik D. Olson[1,2,3,4,^] and Karin Musier-Forsyth[1,2,3,4,*]

Table S1: Table of RNA and DNA primer sequences used in this study.

RNA	Sequence (5'-3')
tRNA^{Lys3}	GCCCGGATAGCTCAGTCGGTAGAGCATCAGACTTTTAATCTG<br>AGGGTCCAGGGTTCAAGTCCCTGTTCGGGCGCCA
PBS/TLE$_{105}$	GGGCUCUGGUAACUAGAGAUCCCUCAGACCCUUUUAGUCA<br>GUGUGGAAAAUCUCUAGCAGUGGCGCCCGAACAGGGACUU<br>GAAAGCGAAAGUAAAGCCAGAGCCC
UTR$_{240}$	UGUGCCCGUCUGUUGUGUGACUCUGGUAACUAGAGAUCCC<br>UCAGACCCUUUUAGUCAGUGUGGAAAAUCUCUAGCAGUGG<br>CGCCCGAACAGGGACUUGAAAGCGAAAGUAAAGCCAGAGG<br>AGAUCUCUCGACGCAGGACUCGGCUUGCUGAAGCGCGCAC<br>GGCAAGAGGCGAGGGGCGGCGACUGGUGAGUACGCCAAA<br>AAUUUUGACUAGCGGAGGCUAGAAGGAGAGAGAUGGGUGC<br>G
UTR$_{240}$(ΔDIS)	UGUGCCCGUCUGUUGUGUGACUCUGGUAACUAGAGAUCCC<br>UCAGACCCUUUUAGUCAGUGUGGAAAAUCUCUAGCAGUGG<br>CGCCCGAACAGGGACUUGAAAGCGAAAGUAAAGCCAGAGG<br>AGAUCUCUCGACGCAGGACUCGGCUUGCUGGAGACGGCAA<br>GAGGCGAGGGGCGGCGACUGGUGAGUACGCCAAAAAUUUU<br>GACUAGCGGAGGCUAGAAGGAGAGAGAUGGGUGCG
UTR$_{240}$(ΔDIS,ΔPBS)	UGUGCCCGUCUGUUGUGUGAGAGAUCUCUCGACGCAGGA<br>CUCGGCUUGCUGGAGACGGCAAGAGGCGAGGGGCGGCGA<br>CUGGUGAGUACGCCAAAAAUUUUGACUAGCGGAGGCUAGA<br>AGGAGAGAGAUGGGUGCG
antiPBS$_{18}$ (DNA)	GTCCCTGTTCGGGCGCCA
antiPBS$_{18}$+3 (DNA)	GTCCCTGTTCGGGCGCCACTG
antiPBS$_{18}$+6 (DNA)	GTCCCTGTTCGGGCGCCACTGCTA
antiPBS$_{18}$+11 (DNA)	GTCCCTGTTCGGGCGCCACTGCTAGAGAT
SHAPE/XL RT primer (DNA)	GGCGCCCATCTCTCTCCTTC

PBS/TLE$_{105}$	X	X	X	X	X
antiPBS$_{18}$					X
antiPBS$_{18}$+3				X	
antiPBS$_{18}$+6			X		
antiPBS$_{18}$+11		X			

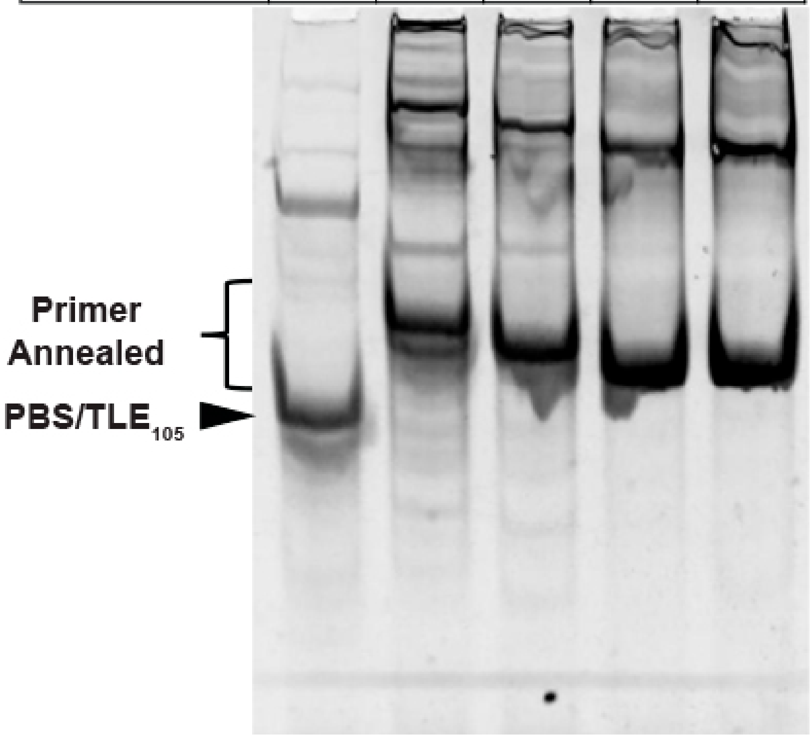

Figure S1: Native PAGE analysis of DNA-primer-annealed PBS/TLE complexes. A 12% native polyacrylamide gel visualized by ethidium bromide staining shows complete annealing of antiPBS DNA oligonucleotides (1.5 molar excess) to PBS/TLE$_{105}$ RNA. This gel was prepared with Tris-Borate buffer supplemented with 1 mM $MgCl_2$ and was run at 120V at 4 ˚C.

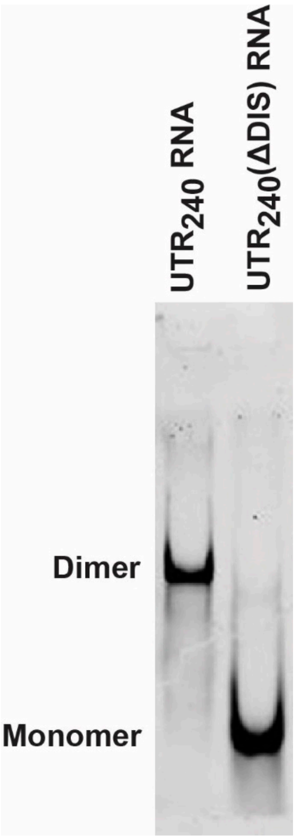

Figure S2: Native PAGE analysis of UTR_{240} RNAs. A 6% native polyacrylamide gel visualized by ethidium bromide staining, shows the WT UTR_{240} RNA runs as a dimer and the UTR_{240}(ΔDIS) RNA runs as a monomer. This gel was prepared with Tris-Borate buffer supplemented with 1 mM $MgCl_2$ and was run at 100V at 4 ˚C.

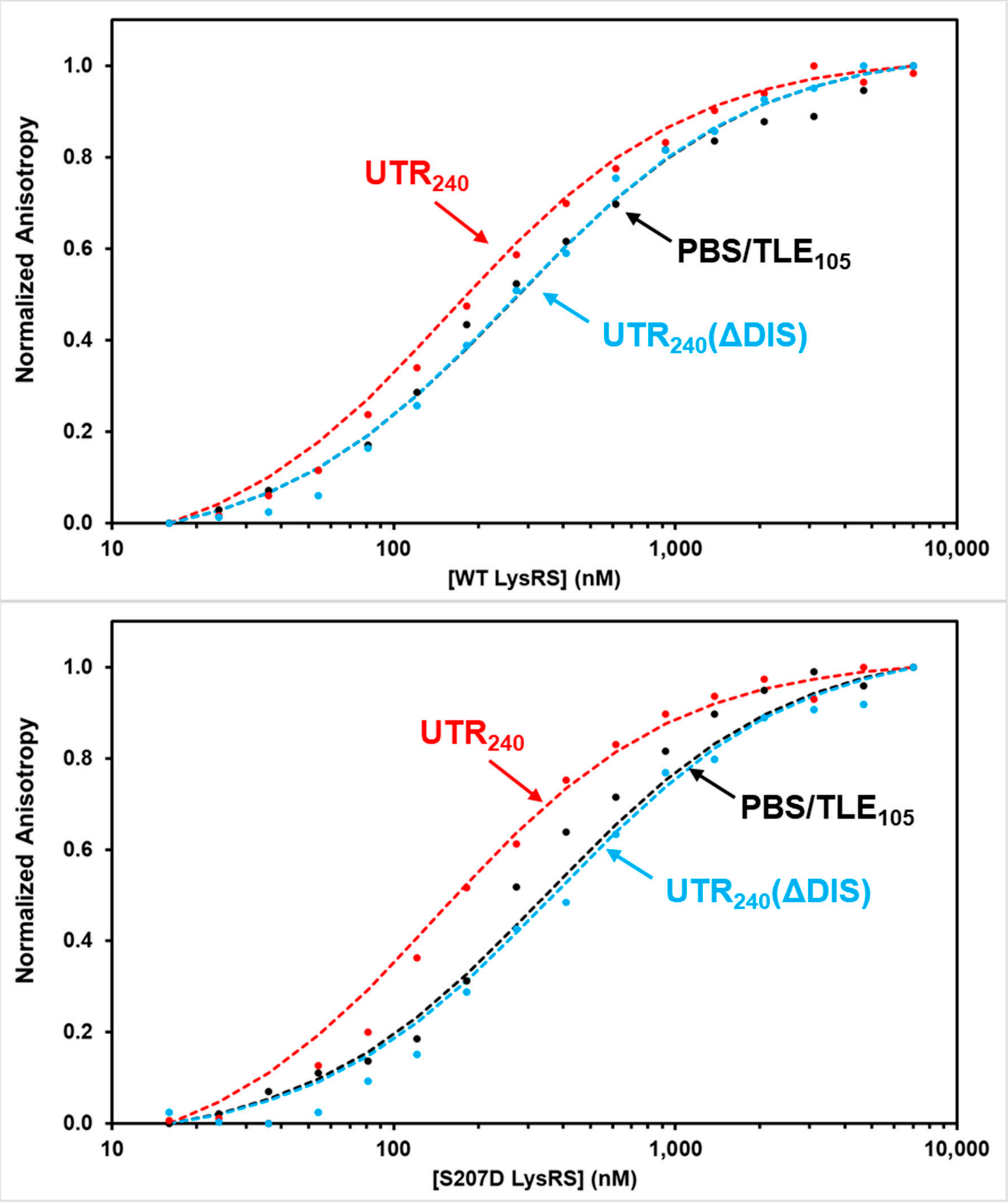

Figure S3: Fluorescence anisotropy plots of WT and S207D LysRS(∆N65) binding to HIV-1 RNAs. All measurements were performed with 30 nM RNA in 15 mM NaCl, 35 mM KCl, 20 mM Tris-HCl pH 8, and 1 mM $MgCl_2$. Results are the average of at least three trials are plotted as the normalized anisotropy as a function of LysRS(∆N65) concentration. Dots denote the average data with dashed lines representing the fitted anisotropy curve fit.

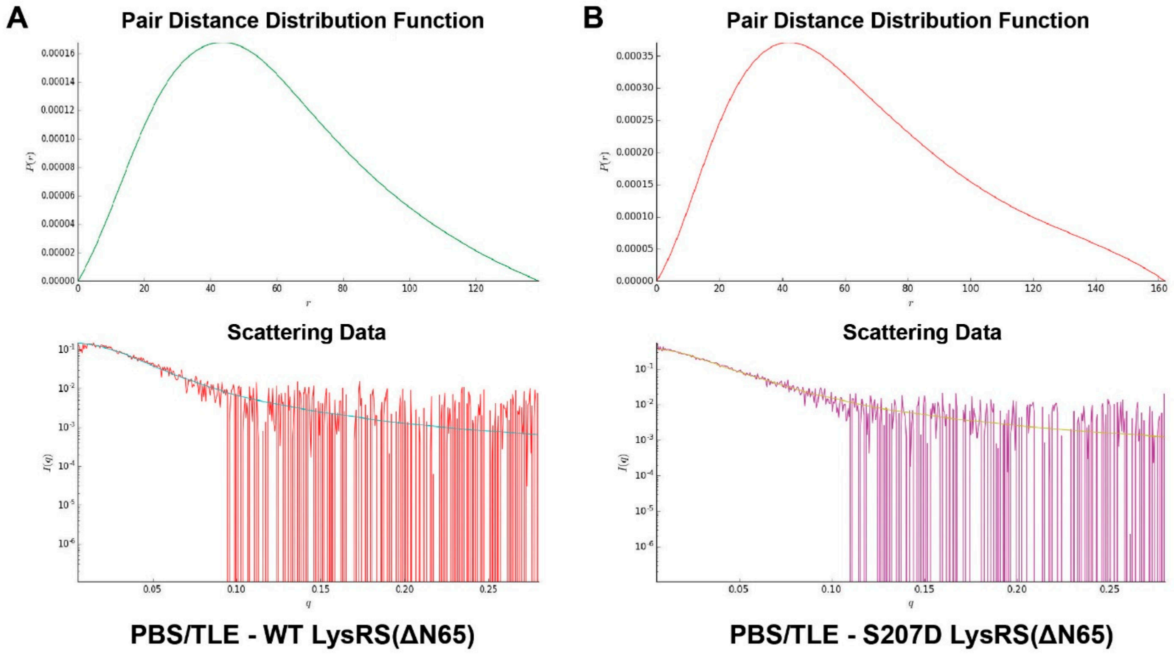

Figure S4: SAXS processing of LysRS-bound PBS/TLE. Pair distance distribution function (top) and raw scattering data (bottom) for PBS/TLE bound to (A) WT LysRS(ΔN65) and (B) S207D LysRS(ΔN65). Overlaid within the raw data plots are the fit lines (smooth lines) calculated from the pair distance distribution plots.

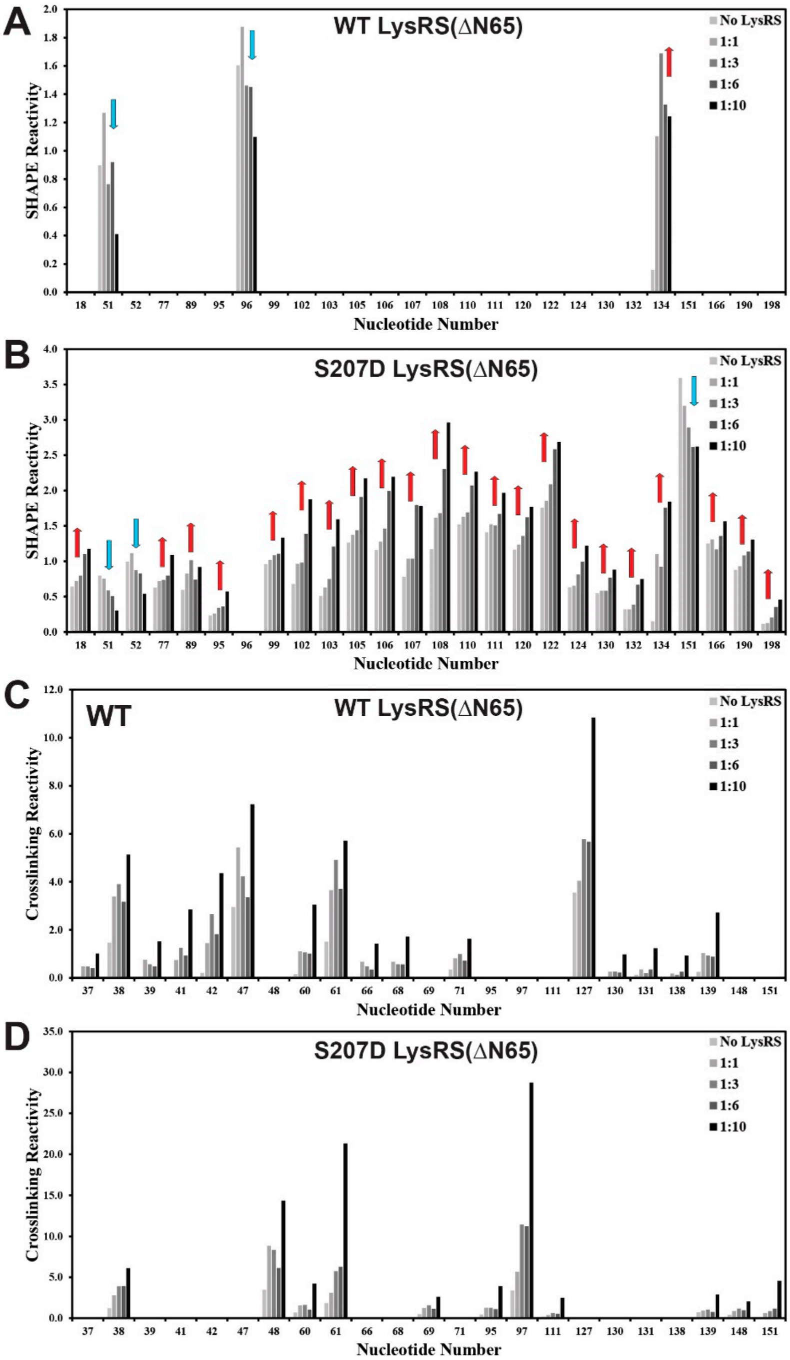

Figure S5: Results of XL-SHAPE titration of LysRS constructs binding to UTR_{240} RNA. SHAPE and XL reactivity changes are plotted with increasing RNA:protein ratio depicted as lighter bars going to darker bars. Only those reactivity changes that were determined to be both dose-dependent and with a magnitude from 'No LysRS' to '1:10 ratio' of > 0.3 were plotted here and in Figure 3. The average of at least three independent experiments were plotted for (A) WT and (B) S207D LysRS(DN65) SHAPE reactivity changes and (C) WT and (D) S207D LysRS(DN65)

crosslinking reactivity changes. For plots (A) and (B), increased and decreased reactivity changes are denoted by red-up and blue-down arrows, respectively.

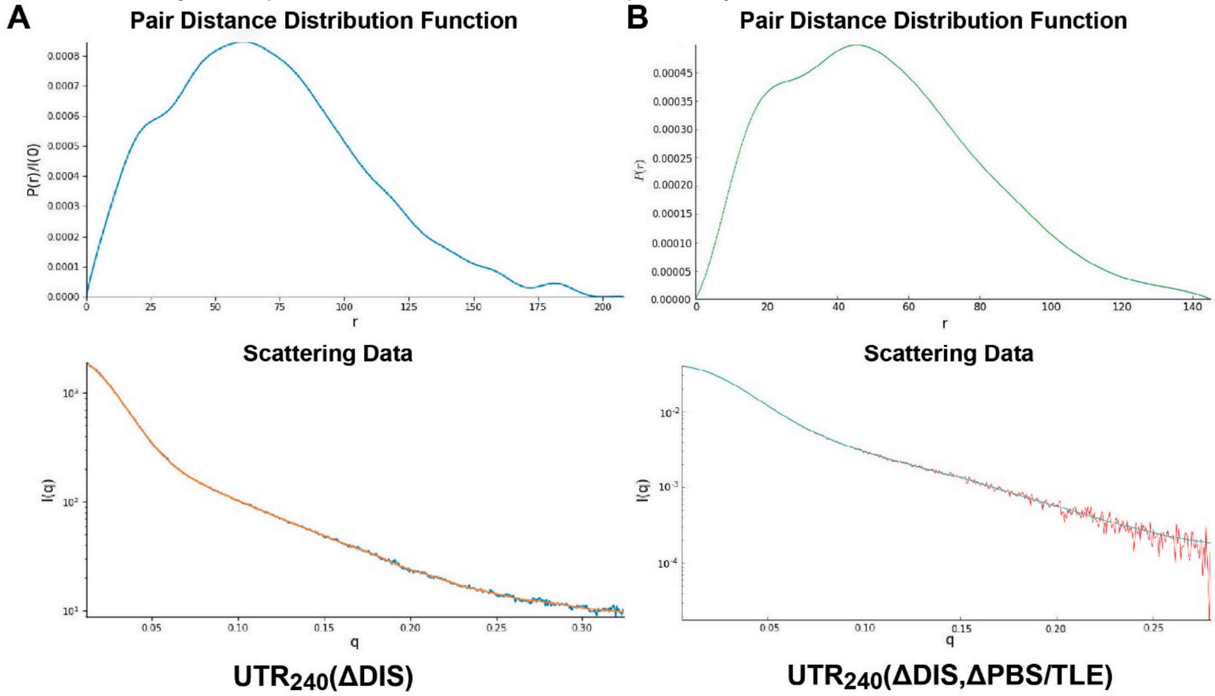

Figure S6: SAXS processing of UTR_{240}(ΔDIS) and UTR_{240}(ΔDIS,ΔPBS/TLE) constructs. Pair distance distribution function (top) and raw scattering data (bottom) for (A) UTR_{240}(ΔDIS) and (B) UTR_{240}(ΔDIS,ΔPBS/TLE). Overlaid within the raw data plots are the fit lines (smooth lines) calculated from the pair distance distribution plots.

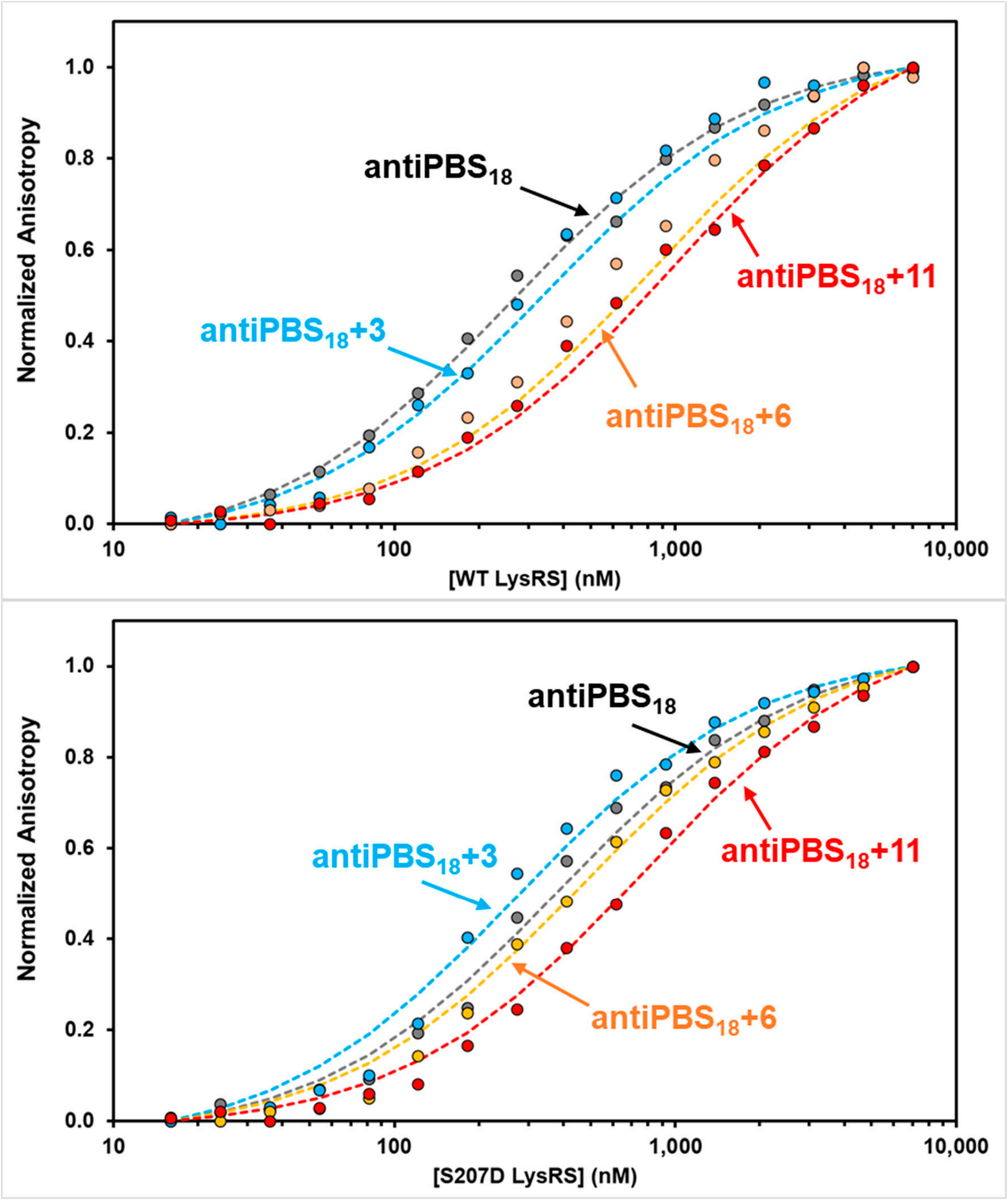

Figure S7: Fluorescence anisotropy plots of WT and S207D LysRS(ΔN65) binding to primer-annealed HIV-1 PBS/TLE$_{105}$ RNAs. All measurements were performed with 30 nM RNA in 15 mM NaCl, 35 mM KCl, 20 mM Tris-HCl pH 8, and 1 mM $MgCl_2$. Results are the average of at least three trials are plotted as the normalized anisotropy as a function of LysRS(ΔN65) concentration. Dots denote the average data with dashed lines representing the fitted anisotropy curve fit.

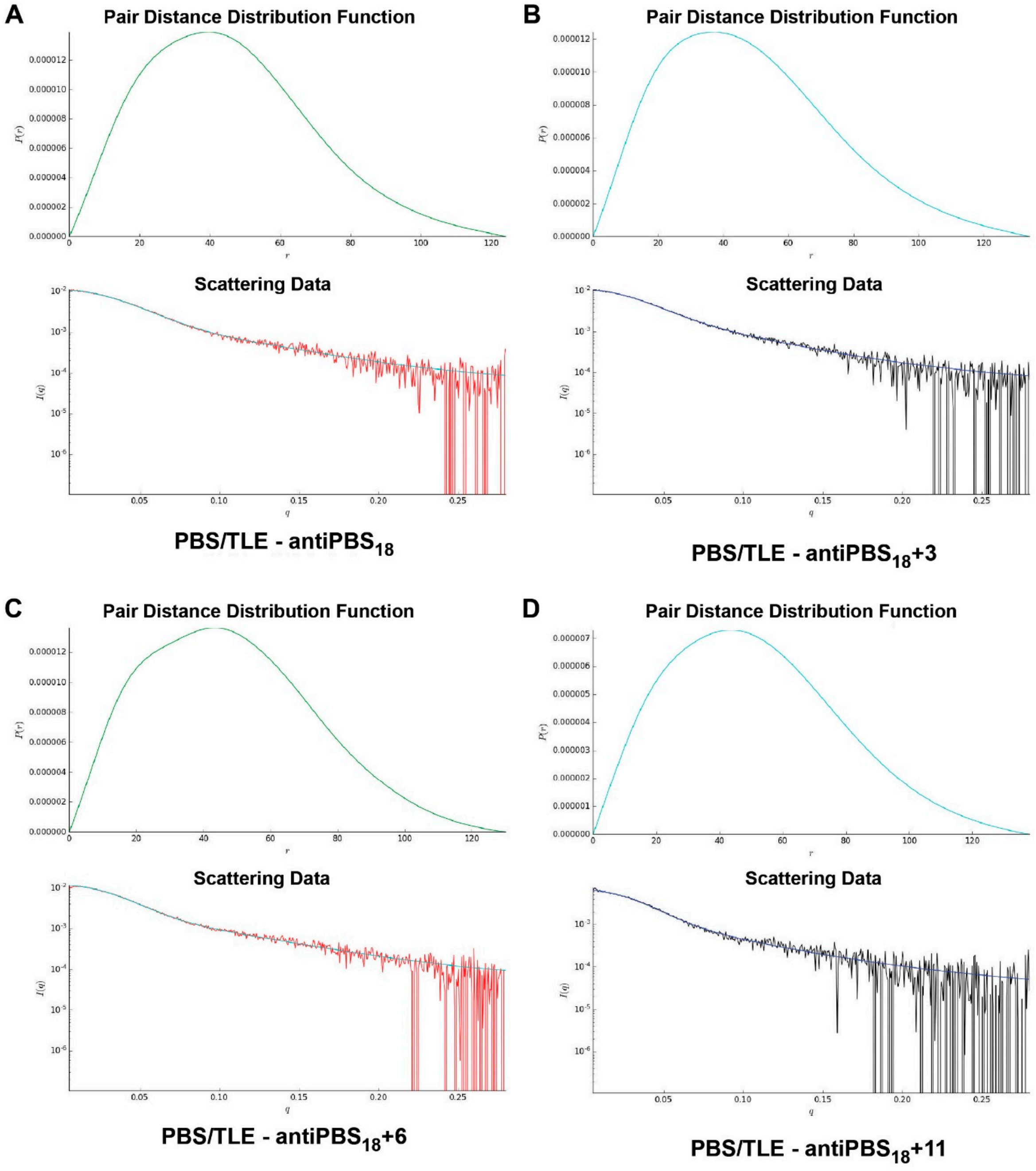

Figure S8: SAXS processing of DNA-primer annealed PBS/TLE. Pair distance distribution function (top) and raw scattering data (bottom) for PBS/TLE annealed by (A) antiPBS$_{18}$, (B) antiPBS$_{18}$+3, (C) antiPBS$_{18}$+6 and (D) antiPBS$_{18}$+11. Overlaid within the raw data plots are the fit lines (smooth lines) calculated from the pair distance distribution plots.

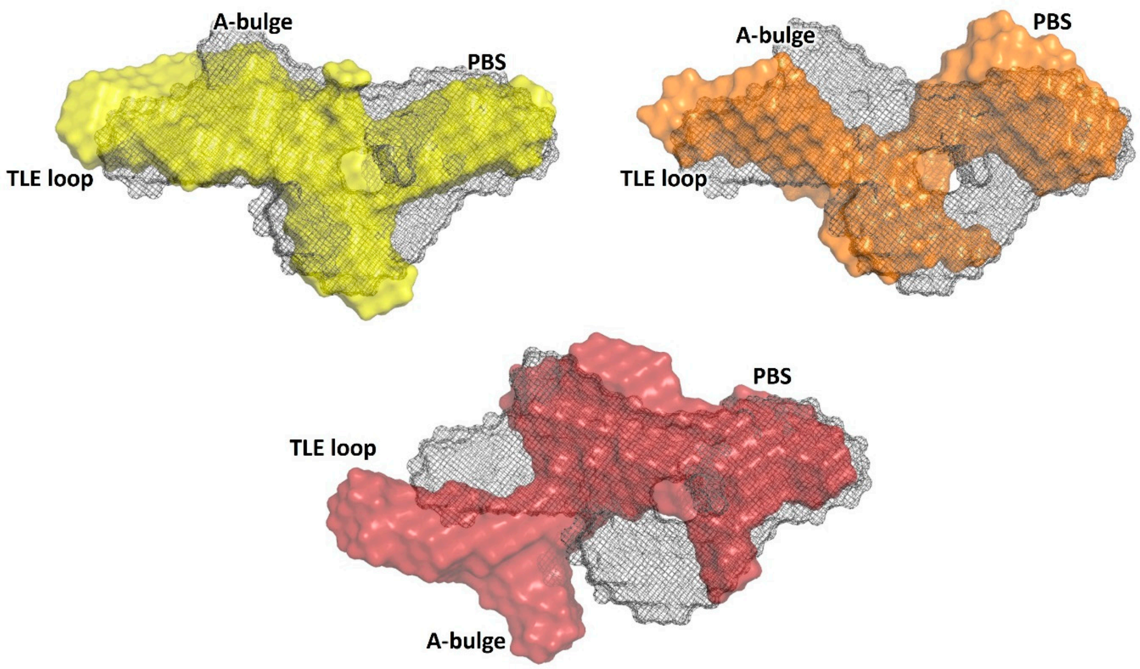

Figure S9: Superposed orthogonal views of all primer-annealed HIV-1 PBS/TLE$_{105}$ RNA SAXS-derived *ab initio* envelopes. All SAXS-derived *ab initio* envelopes were superposed using DAMSUP from the ATSAS software package (as described in the methods). PBS/TLE$_{105}$ annealed to antiPBS$_{18}$ (gray mesh) is superposed with PBS/TLE$_{105}$ annealed to each of antiPBS$_{18}$+3 (yellow surface, top left), antiPBS$_{18}$+6 (orange surface, top right) and antiPBS$_{18}$+11 (red surface, bottom).
